# Longitudinal Improvement of Neurocognition after Electroconvulsive Therapy in Bipolar Depression

**DOI:** 10.1155/2021/7748073

**Published:** 2021-08-16

**Authors:** Tomosuke Nakano, Eishi Motomura, Toshiki Hasegawa, Yasuhiro Kawano, Motohiro Okada

**Affiliations:** Department of Neuropsychiatry, Mie University Graduate School of Medicine, 2-174 Edobashi, Tsu 514-8507, Japan

## Abstract

Electroconvulsive therapy (ECT) is applied to treatment-resistant mood disorders. Its therapeutic effect on neurocognition remains unclear. We report the case of a 55-year-old man with treatment-resistant bipolar depression who underwent ECT series. We longitudinally monitored his neurocognition with the Brief Assessment of Cognition in Schizophrenia-Japanese version (BACS-J). The patient's scores on all of the BACS-J domains except working memory recovered after the ECT series. Interestingly, his verbal memory, motor speed, and executive function recovered 1 month after ECT, whereas his verbal fluency and attention scores recovered approx. 1 year after ECT. The BACS can be useful for monitoring ECT's longitudinal effects on individuals' cognitive recovery. Further studies with a large sample size are needed to confirm our present findings.

## 1. Introduction

Electroconvulsive therapy (ECT) is effective for the treatment of individuals with antidepressant-resistant major depression (for guidelines, see [1]) and those with mood stabilizer-resistant bipolar disorder [2]. Cognitive impairment is a clinically important treatment target, but the effects of ECT on neurocognition have been unclear. The Brief Assessment of Cognition in Schizophrenia (BACS) is designed to assess multiple aspects of neurocognitive functioning in individuals with schizophrenia [3]. Here, we describe the therapeutic effect of ECT on neurocognition evaluated by the BACS-J (Japanese version) [4] in a patient with treatment-resistant bipolar depression. Written informed consent for his case to be published was obtained from the patient.

## 2. Case Presentation

A 55-year-old man who had been treated with several antidepressants was admitted to our ward with a refractory depressive episode. At the first medical examination, he presented depressive mood, fatigue, and negative thoughts. He could not sit calmly, due to agitation. There was a familial history of depression in his brother. No psychiatric history was reported for him. During that admission, the patient presented accelerated speech with elevated mood, increasing energy, grandiosity, and irritability for several days. Based on the Diagnostic and Statistical Manual Disorders, 5th edition [5], he was rediagnosed with bipolar II disorder (DSM-5 code: 296.89). After this diagnosis, the patient's antidepressant treatment was switched to several mood stabilizers and antipsychotics, including quetiapine (300 mg/day) and valproate (1200 mg/day). However, his depressive mood and agitation did not improve sufficiently.

**Figure 1:**
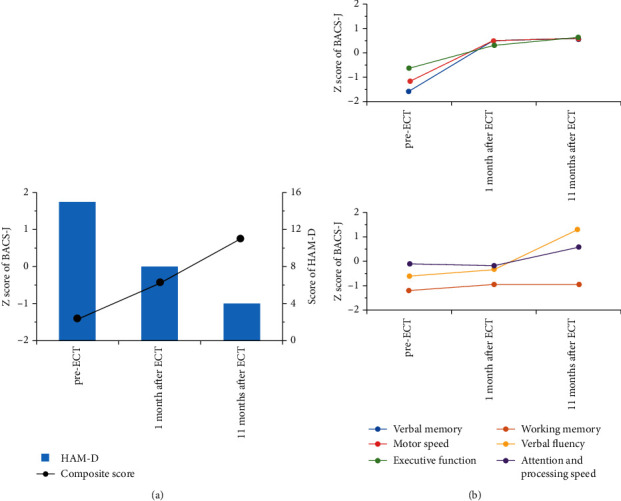
The patient's HAM-D and BACS-J scores at pre- and post-mECT. (a) The total scores on the HAM-D and the composite *Z*-scores on the BACS-J. (b) Each subdomain *Z*-score was calculated using the means and standard deviations based on data obtained from healthy controls [16]. The composite score was calculated by averaging all *Z*-scores obtained from the six subdomains of the BACS-J.

He gradually became exhausted due to his prolonged (>2-year) depression and then exhibited suicidal ideation. At his present admission, the results of blood tests, electroencephalography, and MRI were normal. To prevent his suicidal behavior, a total of 10 sessions of modified ECT (mECT) was carried out under anesthesia and muscle-relaxant medications. During the mECT course, quetiapine (300 mg/day) was continued, but valproate was discontinued. After the patient completed this mECT, his depressive symptoms improved considerably and suicidal thoughts disappeared. He was treated with the daily maintenance medication of quetiapine 200 mg and valproate 600 mg (serum level 44.1 mg/L). His scores on both the Hamilton Rating Scale for Depression (HAM-D) and the BACS-J recovered (Figure 1(a)). At 1 month after the patient completed the mECT series, his scores on three domains of BACS-J (verbal memory, motor speed, and executive function) had recovered (Figure 1(b)). About 1 year after the mECT series, the patient's scores on the remaining two domains of BACS-J (verbal fluency and attention and processing speed) showed delayed improvement; however, his working memory score had not improved.

He was discharged 2 months after the mECT series and was followed in outpatient settings. Along with the neurocognitive improvement, he achieved a recovery of depressive symptoms and daily/social function that allowed him to begin working again.

## 3. Discussion

To the best of our knowledge, this is the first reported case in which a patient's cognitive improvement was longitudinally evaluated after mECT by using the BACS-J. A learning effect of the BACS-J used in this case has been described; Kaneda et al. [4] reported the good test-retest reliability of BACS-J by using two different versions within a short interval. We speculate that there is less possibility of a learning effect in our patient's case because (1) the intervals between the assessment were >1 month and (2) two different versions of the BACS-J were administered alternatively in three sessions.

Several recent studies described after ECT improvements in patients' cognitive function over long-term follow-up periods [6–8]. In our patient, compared to his scores on the BACS-J before the mECT series, the composite BACS-J score and the scores on all of the BACS-J domains except for working memory recovered along with his depressive symptoms after his treatment with mECT, which resulted in his social recovery. Interestingly, three domains improved immediately (verbal memory, motor speed, and executive function), but others improved later.

Anderson et al. [6] reported that patients with remission after mECT showed significant improvements in verbal fluency at 4 months after mECT compared to unremitted patients. However, our patient's working memory deficits persisted during a 1-year follow-up. A systematic review suggested that the working memory deficit persists even in remitted patients with bipolar disorder [9].

Several test batteries have been used after mECT cognitive assessments, including the Montreal Cognitive Assessment (MoCA), the Mini-Mental State Examination (MMSE), and the Measurement and Treatment Research to Improve Cognition in Schizophrenia (MATRICS) Consensus Cognitive Battery (MCCB). The MoCA and MMSE were developed as a tool to screen dementia patients. They can be easily administered within ≤10 min, whereas the disadvantage of these batteries is their “ceiling effect.” In contrast, the MCCB was developed primarily for the assessment of neurocognition in individuals with schizophrenia disorders and has been widely used for other patients with psychiatric disorders. The administration of the MCCB takes ≥60 min. The BACS can be useful for longitudinally monitoring the neurocognition of patients treated with mECT, as it can be completed noninvasively within approx. 30 min and can monitor each domain of cognition separately.

In addition to these traditional cognitive properties, a focus on emotional characteristics of patients with mood disorders is needed. The Brief Assessment of Cognition in Affective Disorder (BAC-A) was developed as a comprehensive cognitive battery [10], but the suitability of the BAC-A for patients with bipolar depression remains to be explored. Moreover, the BAC-A has not been translated into Japanese. These are the reasons why we used the BACS-J in the present case.

Several studies revealed that there was no significant difference in cognition between patients in a euthymic or noneuthymic state [11, 12]; that is, unlike our patient's case, the improvement of depressive symptoms is not associated with the improvement of cognition. In clinical settings, we should focus on the patients' cognition as well as the severity of their illness. We propose that it is important to monitor longitudinal improvements of patients' neurocognition after ECT.

Medications may also affect cognition. Indeed, quetiapine improves discriminability and the performance of a learning task, short-term task, and recognition tasks in patients with bipolar disorder [13, 14]. Valproate might have an adverse effect on cognition—especially working memory—in individuals with bipolar disorder [15]. Further studies with large sample sizes are also needed to clarify whether there is a difference in the effects of ECT and medications on cognition during long-term follow-up.

In conclusion, we have described the case of a patient with bipolar depression who showed longitudinal improvements in BACS-J sores along with a recovery of depressive symptoms after mECT. We speculate that the improvement in this patient's neurocognition might have led to his positive prognosis.

## Data Availability

The data used to support the findings of this study are included within the article.
